# Statistical CSI-Based Beamspace Transmission for Massive MIMO LEO Satellite Communications

**DOI:** 10.3390/e27121214

**Published:** 2025-11-28

**Authors:** Qian Dong, Yafei Wang, Nan Hu, Yiming Zhu, Wenjin Wang, Li Chai

**Affiliations:** 1 National Mobile Communications Research Laboratory, Southeast University, Nanjing 210096, China; 220250853@seu.edu.cn (Q.D.); wangyf@seu.edu.cn (Y.W.); ymzhu@seu.edu.cn (Y.Z.); 2 Purple Mountain Laboratories, Nanjing 211100, China; 3 China Mobile Research Institute, Beijing 100032, China; chaili@chinamobile.com

**Keywords:** LEO satellite, precoding, statistical CSI, massive MIMO, beam selection

## Abstract

In multibeam low-Earth-orbit (LEO) satellite systems, precoding has emerged as a key technology for mitigating co-channel interference (CCI) and for improving spectral efficiency (SE). However, its practical implementation is challenged by the difficulty of acquiring reliable instantaneous channel state information (iCSI) and by the high computational complexity induced by large-scale antenna arrays, making it incompatible with fixed codebook-based beamforming schemes commonly adopted in operational systems. In this analysis, we propose a beamspace transmission framework leveraging statistical CSI (sCSI) and achieves reduced computational complexity compared with antenna-domain precoding designs. Specifically, we first propose a low-complexity beam selection algorithm that selects a small subset of beams for each user terminal (UT) from a fixed beamforming codebook, using only the UTs’ two-dimensional (2D) angular information. To suppress CCI among beams, we then derive a beamspace weighted minimum mean square error (WMMSE) precoding scheme based on the equivalent beamspace channel matrix. The derivation employs an sCSI-based WMMSE (sWMMSE) formulation derived from an upper bound approximation of the ergodic sum rate, which provides a tighter estimate than the expected mean square error (MSE)-based lower bound approximation. Simulation results demonstrate that the proposed sCSI-based beamspace transmission scheme achieves a favorable trade-off between performance and computational complexity.

## 1. Introduction

With the advent of 5G and beyongd-5G networks, ubiquitous connectivity has become a fundamental requirement [1]. Although terrestrial networks have witnessed remarkable advances, vast oceanic regions and sparsely populated rural areas remain underserved, either because the deployment of terrestrial base stations is infeasible or not economically viable [2,3]. Satellite communications have emerged as a promising solution, as their integration with terrestrial networks enables seamless coverage regardless of geographical and topographical limitations. In particular, low-Earth-orbit (LEO) satellite communication systems, characterized by lower propagation delay and reduced deployment costs compared with medium-Earth-orbit (MEO) and geostationary (GEO) systems, are especially suitable for meeting the growing communication demands that require low latency and cost efficiency [4,5].

Whereas the satellite coverage area hosts a large number of user terminals (UTs), on-board resources such as spectrum and transmit power remain highly constrained, rendering the traditional single broad-beam coverage inadequate to satisfy practical requirements [3,6]. To cope with this limitation, advanced techniques from terrestrial networks, such as massive multiple-input multiple-output (mMIMO) and fully digital beamforming (DBF) architecture, have been introduced into LEO satellite communication systems. By directing multiple spot beams to different UTs and leveraging frequency or polarization reuse, these systems achieve substantial gains in spectral efficiency (SE) and system throughput [2].

To achieve multibeam transmission for LEO satellites with minimal complexity, the fixed-beam beamforming method based on codebooks is commonly employed. Ref. [7] proposed a Discrete Fourier Transform (DFT)-based beamforming algorithm, while [8,9] further reduce implementation costs by leveraging two-dimensional (2D) Fast Fourier Transform (FFT) techniques. These approaches enable the generation of multiple independent beams with significantly low computational consumption. However, their lack of beam-steering flexibility presents drawbacks, hindering the system from adapting to irregular or dynamic traffic distributions [10,11]. Additionally, since aggressive full-frequency reuse (FFR) is applied across beams to improve SE and provide higher bandwidth to UTs, this inevitably results in severe co-channel interference (CCI) [12,13]. It is worth noting that, as UTs deviate from the steering center of beams generated by a fixed codebook, they may experience significant CCI with multiple active UTs nearby. The fixed-beam beamforming technique, however, fails to effectively mitigate such interference, which severely constrains the system performance.

A common strategy to mitigate such interference is to perform linear precoding (LP) at the transmitter, where the signals are processed based on the estimated channel statement information (CSI) [2,13,14,15,16,17,18,19,20]. By fully exploiting the spatial freedom offered by satellite phased arrays and suppressing CCI, the system capacity can be effectively enhanced. However, obtaining accurate instantaneous CSI (iCSI) in LEO satellite communication systems is inherently challenging, as the channels vary rapidly due to the high mobility of the LEO satellite and the feedback resources are limited [2]. To this end, many works have considered the impact of CSI acquisition errors on precoding performance and proposed corresponding robust precoding methods, among which statistical channel state information (sCSI), e.g., angle of departure (AoD) and channel statistics, has been widely employed [15]. For example, ref. [16] designed a robust precoding scheme under per-antenna power constraints by accounting for imperfect angle-of-departure (AoD) information, with the objective of maximizing the system sum rate. Ref. [17] utilized sCSI in precoding design to enhance the average signal-to-leakage-plus-noise ratio (SLNR). Moreover, refs. [18,19,20] exploited sCSI to implement classical matched filter (MF), zero-forcing (ZF), and regularized zero-forcing (RZF) precoding methods, respectively.

Despite their excellent performance in suppressing interference and improving robustness, these precoding schemes for LEO satellite communication systems are typically anchored in the antenna-domain, where high-dimensional matrix operations are still required, thus imposing substantial computational complexity [21]. To alleviate the computational burden on practical on-board processor (OBP) payloads, the exploration of low-complexity beamspace precoding approaches is motivated, where lower-dimensional beamspace channels are exploited. Refs. [10,22] implemented LP methods over beamspace channels. Specifically, ref. [10] proposed an iterative algorithm that iteratively optimizes beam selection and beamspace precoding design by utilizing the Hungarian method and weighted minimum mean square error (WMMSE) precoding, while [22] considered coordinated multiple-satellite (CoMSat) systems and developed an iterative algorithm to jointly optimize beam selection and LP with the objective of minimizing the total transmit power. However, these studies mostly assume perfect iCSI, whereas the slowly varying sCSI-based transmission, which offers a more practical solution for real deployments, remains neglected.

Based on the above analysis, to the best of our knowledge, no prior work has performed beamspace LP in conjunction with exploiting sCSI for LEO satellite downlink transmissions to overcome both the challenges of iCSI acquisition and reduce the computational burden of antenna-domain precoding design. Motivated by this gap, in this paper, we explore a low-complexity yet efficient sCSI-based downlink multibeam transmission scheme for a LEO satellite communication system. In summary, our main contributions are as follows:

- We propose an sCSI-based multibeam transmission framework. Specifically, we first select beams for UTs from a fixed beamforming codebook, then perform LP based on the equivalent beamspace channel. To fully exploit the sCSI for performance optimization, we analyze the sCSI-based upper and lower bound approximations of the ergodic sum rate and show that the upper bound offers a tighter estimate. Based on this approximation, we formulate the weighted sum rate (WSR) maximization problem, subject to constraints on the power budget and the maximum number of simultaneously activated beams.
- We propose an angle-based beam selection algorithm that efficiently selects beams from a fixed codebook. To improve the beamspace representation of the antenna-domain channel, we evaluate the normalized beamforming gain of each beam toward a given UT and assign at least one beam to each UT. In addition, we simplify the beam selection process and reduce feedback overhead by reformulating the beamforming gain as a function of the beam’s angular offset from the UT’s line-of-sight (LoS) direction. Simulation results demonstrate that, compared with the baseline scheme that selects a single beam best aligned with each UT’s LoS direction, the proposed algorithm achieves improved sum rate performance.
- Based on the equivalent beamspace channel, we reformulate the WSR maximization problem as a WMMSE problem through covariance decomposition and derive an sCSI-based WMMSE (sWMMSE) precoding scheme. The proposed beamspace precoding effectively lowers computational complexity compared with antenna-domain schemes, as it operates on a reduced-dimensional beamspace channel matrix. Simulation results show that the proposed sWMMSE precoding scheme converges rapidly within only a few iterations.

The remainder of this paper is organized as follows. Section 2 introduces the system and channel models. Section 3 presents the proposed two-stage beamspace transmission design, including the angle-based beam selection and the beamspace WMMSE precoding algorithm, both relying only on sCSI. Section 4 provides simulation results and performance comparisons with benchmark schemes. Section 5 concludes the paper.

*Notation*: x,x,X represent scalar, column vector, and matrix. ${\left(\cdot \right)}^{T}$, ${\left(\cdot \right)}^{H}$, and ${\left(\cdot \right)}^{-1}$, respectively, denote the transpose, transpose-conjugate, and inverse operations. $I_{M}$ represents M×M identity matrix. ${∥\cdot ∥}_{2}$ denotes $l_{2}$-norm. ⊗ is the Kronecker product operations. The operator Tr(·) represents the matrix trace. diag{a} represents the diagonal matrix whose diagonal elements are composed of a. ${\left[X\right]}_{i,j}$ and ${\left[x\right]}_{i}$ denotes the (i,j)-th and *i*-th element of x and X, respectively. x⪰0 means all the elements of x is non-negative. The expression $CN(\mu ,\sigma^{2})$ denotes circularly symmetric Gaussian distribution with expectation μ and variance $\sigma^{2}$. $C^{M\times N}$ and $Z^{M\times N}$ represent the set of M×N dimension complex- and integer-valued matrixes.

## 2. System Model

### 2.1. System Setup

**Figure 1:**
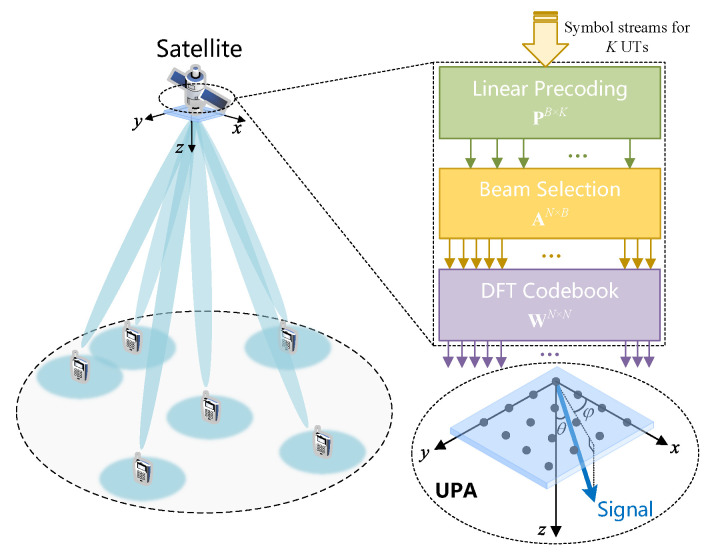
System model.

As illustrated in Figure 1, we consider an OBP payload-enabled LEO satellite that serves *K* single-antenna UTs within its coverage area. Let K=1,2,…,K denote the set of all UTs. The satellite is equipped with a uniform planar array (UPA) consisting of $M=M_{x}M_{y}$ antennas and adopts a fully digital precoding architecture to support multibeam transmission. Owing to the on-board power constraint, at most, B≥K digital beams can be simultaneously activated. To maximize spectral efficiency, FFR is adopted across beams. At a given symbol time, the transmit signal for *K* UTs denoted as $s={\left[s_{1},s_{2},\ldots ,s_{K}\right]}^{T}\in C^{K\times 1}$ is generated by the modulation and coding module, satisfying $E\left[ss^{H}\right]=I_{K}$.

The satellite OBP then processes this signal through two core functional modules. The first is the LP module, which applies the precoding matrix $P=\left[p_{1},\ldots ,p_{K}\right]\in C^{B\times K}$ to the symbol vector s, thereby steering beams and suppressing multiuser interference so that UTs can share the same time–frequency resources. With massive arrays but limited on-board computation, LP methods significantly reduce complexity relative to nonlinear schemes while maintaining strong performance. In line with the satellite’s stringent power budget, the precoder is designed under a total transmit power constraint

(1) $$\left(C1\right):\;\mathrm{Tr}\left(PP^{H}\right)\leq P_{T}.$$

The second is the beam selection module. Transmit beamforming is implemented by selecting beams from a DFT-based beamforming codebook $W=\left[w_{1},\ldots ,w_{N}\right]\in C^{M\times N}$, where N>K denotes the number of codewords that can be selected form the codebook and each column $w_{n}$ is a codeword. Denote N={1,2,…,N} as the index set of codewords in W. We construct the codebook W and determine *N* as follows. First, we generate a fully dimensional DFT-based beamforming codebook $W^{\mathrm{DFT}}\in C^{M\times M}$ by applying a 2D-DFT to the antenna array. Then, since some beams generated from $W^{\mathrm{DFT}}$ point outside the satellite’s maximum coverage area, we exclude the codewords corresponding to these infeasible beams, yielding an reduced-dimensional codebook $W\in C^{M\times N}$.

Let the precoded symbol vector be x=Ps. We introduce a binary codeword-to-beam assignment matrix $A\in {\left\{0,1\right\}}^{N\times B}$; then, its (n,b)-th entry $a_{n,b}$ indicates whether codeword $w_{n}$ is used to synthesize the *b*-th transmit beam

(2) $$a_{n,b}=\left\{\begin{array}{cc} 1, & \mathrm{if}\;\mathrm{codeword}\;w_{n}\;\mathrm{is}\;\mathrm{assigned}\;\mathrm{to}\;x_{b},\\ 0, & \mathrm{otherwise}. \end{array}\right.$$

The assignment matrix A must satisfy the following constraints:

(3) $$\begin{array}{cc} (C2):\; & \sum_{n=1}^{N}a_{n,b}\leq 1,\;\forall b,\\ (C3):\; & \sum_{b=1}^{B}a_{n,b}\leq 1,\;\forall n. \end{array}$$

Equivalently, (C2) and (C3) enforce a one-to-one matching between a subset of codewords and the available beam indices. Since A has *B* columns, the number of active beams equals the number of nonzero columns of A and is therefore upper bounded by *B*.

Based on the system model described above, the signal received by UT *k* can be written as

(4) $$y_{k}=h_{k}^{H}WAPs+n_{k},$$

where $h_{k}$ denotes the downlink channel vector from the satellite antenna array to UT *k*, and $n_{k}$ is additive noise following $\mathrm{CN}(0,\sigma_{k}^{2})$.

According to (4), the transmitted signal is denoted by

(5) $$x^{A}=WAx^{B}=WAPs,$$

where $x^{B}=Ps\in C^{B\times 1}$ denotes the transmitted signal in the beamspace domain, whose corresponding beamspace channel is $A^{H}W^{H}h_{k}\in C^{B\times 1}$ for UT *k*. Similarly, $x^{A}=WAx^{B}\in C^{M\times 1}$ represents the transmitted signal in the antenna-domain, with its corresponding antenna-domain channel being $h_{k}\in C^{M\times 1}$.

The difference between the antenna-domain transmit signal $x^{A}$ and the beamspace transmit signal $x^{B}$ essentially lies in their corresponding channel representations. After selecting *B* beams from the predefined codebook, the satellite forms *B* outgoing beams, each pointing toward a different spatial direction. Consequently, each UT’s antenna-domain channel vector is projected onto these spatial directions, and the resulting projection coefficients correspond to the beamforming gains. This process reduces the original *M*-dimensional antenna-domain channel vector to a *B*-dimensional beamspace representation.

To further clarify the relationship between $x^{A}$ and $x^{B}$, we describe how the latter is transformed into the former and highlight their physical meanings. The *B*-dimensional vector $x^{B}$ represents the signals to be radiated along the *B* beams, where each element corresponds to one beam and specifies which UTs’ signals are carried in the beam and how much power is radiated toward it. After being processed by WA, $x^{B}$ is mapped to the antenna-domain for transmission through the physical antenna array. Since each column of WA characterizes the spatial excitation pattern of one beam across the antenna elements, each element of $x^{B}$ is distributed and weighted across all *M* antennas. Accordingly, the antenna-domain signal $x^{A}$ is formed, with its *n*-th entry corresponding to the baseband signal fed to the *n*-th antenna element.

### 2.2. Channel Model

The downlink channel between the LEO satellite and UT *k* at the instant *t* and frequency *f* can be modeled as [17]

(6) $$h_{k}\left(t,f\right)=\sum_{p=0}^{P_{k}-1}a_{k,p}\;e^{j2\pi \;(t\nu_{k,p}-f\tau_{k,p})}\;v_{k,p}\in C^{M\times 1},$$

where $P_{k}$ represents the number of propagation paths and $a_{k,p}$, $\nu_{k,p}$, $\tau_{k,p}$, $v_{k,p}$ denote the complex gain, Doppler shift, delay, and array response vector of path *p*. When the relative positions of the satellite and UT *k* do not change significantly, $\left\{P_{k},a_{k,p},\nu_{k,p},\tau_{k,p},v_{k,p}\right\}$ can be assumed to be invariant over the time intervals of interest [17]. Specifically, the Doppler shift can be decomposed as $\nu_{k,p}≜\nu_{k}^{\mathrm{sat}}+\nu_{k,p}^{\mathrm{ut}}$, where $\nu_{k}^{\mathrm{sat}}$ refers to a path-independent component caused by the motion of the LEO satellite, and $\nu_{k,p}^{\mathrm{ut}}$ refers to a path-dependent component caused by the motion of UT *k* [23,24]. Let $\tau_{k}^{\min}=\min_{p}\tau_{k,p}$ denote the minimum path delay of UT *k*, then the relative delay of path *p* can be written as $\tau_{k,p}^{\mathrm{ut}}≜\tau_{k,p}-\tau_{k}^{\min}$. Since different elements of the satellite UPA experience phase offsets when transmitting or receiving the same signal, the array response vector is used to capture this characteristic. The array response vector corresponding to the *p*-th propagation path of UT *k* is given by [25]

(7) $$v_{k,p}=v_{k,p}^{x}\left(\vartheta_{k,p}^{x}\right)⊗v_{k,p}^{y}\left(\vartheta_{k,p}^{y}\right),$$

where $\vartheta_{k}^{x}=\sin\left(\theta_{k}\right)\cos\left(\phi_{k}\right)$ and $\vartheta_{k}^{y}=\sin\left(\theta_{k}\right)\sin\left(\phi_{k}\right)$. As illustrated in Figure 1, $\theta_{k}$ and $\phi_{k}$ represent the elevation and azimuth angles of UT *k* with respect to the satellite’s local plane coordinate system, and we denote $\theta_{k}=\left[\theta_{k},\phi_{k}\right]$. The vectors $v_{k,p}^{x}$ and $v_{k,p}^{y}$ denote the array response vectors of the *p*-th propagation path of UT *k* along the *x*-axis and *y*-axis of the satellite UPA, respectively, which can be written as

(8) $$\begin{array}{cc} v_{k,p}^{x} & =\frac{1}{\sqrt{M_{x}}}\;{\left[1,e^{-j\frac{2\pi }{\lambda }d_{x}\vartheta_{k,p}^{x}},\ldots ,e^{-j\frac{2\pi }{\lambda }d_{x}\left(M_{x}-1\right)\vartheta_{k,p}^{x}}\right]}^{T},\\ v_{k,p}^{y} & =\frac{1}{\sqrt{M_{y}}}\;{\left[1,e^{-j\frac{2\pi }{\lambda }d_{y}\vartheta_{k,p}^{y}},\ldots ,e^{-j\frac{2\pi }{\lambda }d_{y}\left(M_{y}-1\right)\vartheta_{k,p}^{y}}\right]}^{T}, \end{array}$$

where $d_{x}$ and $d_{y}$ denote the antenna element spacing of the satellite UPA along the *x*-axis and *y*-axis, which are typically set to $d_{x}=d_{y}=\frac{\lambda }{2}$ to avoid grating lobes. Owing to the considerably higher altitude of the satellite relative to the scatterers around the ground terminals, it is reasonable to assume that, for a given UT, the elevation and azimuth angles of all propagation paths are identical [17], i.e., $\theta_{k,p}=\theta_{k}$ and $\varphi_{k,p}=\varphi_{k}$. Accordingly, we have $\vartheta_{k,p}^{x}=\vartheta_{k}^{x}$, $\vartheta_{k,p}^{y}=\vartheta_{k}^{y}$ and $v_{k,p}=v_{k}$.

Based on the above analysis of the key propagation characteristics in LEO satellite communications, the downlink channel model in (6) can be reformulated as

(9) $$h_{k}\left(t,f\right)=a_{k}\left(t,f\right)\;e^{j2\pi \;(t\nu_{k}^{\mathrm{sat}}-f\tau_{k}^{\min})}\;v_{k},$$

where $a_{k}\left(t,f\right)$ denotes the downlink complex channel gain of UT *k*:

(10) $$a_{k}\left(t,f\right)≜\sum_{p=0}^{P_{k}-1}a_{k,p}\;e^{j2\pi \;(t\nu_{k,p}^{\mathrm{ut}}-f\tau_{k,p}^{\mathrm{ut}})}.$$

### 2.3. Statistical CSI

Due to the LoS-dominated propagation characteristics of LEO satellite communication links, the channel gain term $a_{k}\left(t,f\right)$ in (9) is assumed to follow a Rician distribution with Rician factor $\kappa_{k}$ and average power $\gamma_{k}$. Specifically, the real and imaginary parts of $a_{k}$ are modeled as independent and identically distributed real-valued Gaussian random variables with mean $\sqrt{\frac{\kappa_{k}\gamma_{k}}{2(\kappa_{k}+1)}}$ and variance $\frac{\gamma_{k}}{2(\kappa_{k}+1)}$, respectively [26]. In the LEO satellite communication system, sCSI for satellite UTs includes

(11) $$\begin{array}{cc} {\bar{a}}_{k} & ≜|E\left\{a_{k}\left(t,f\right)\right\}|=\sqrt{\frac{\gamma_{k}\kappa_{k}}{\kappa_{k}+1}},\\ \Omega_{k} & ≜E\left\{h_{k}h_{k}^{H}\right\}=\gamma_{k}\;v_{k}v_{k}^{H}, \end{array}$$

where the rank-one structure of $\Omega_{k}$ captures the dominant LoS spatial signature persisting over multiple coherence blocks.

### 2.4. Problem Formulation

UT *k*’s signal-to-interference-plus-noise ratio (SINR) can be given as

(12) $$\begin{array}{cc} \Gamma_{k}\left(A,P\right)= & \frac{p_{k}^{H}A^{H}W^{H}h_{k}h_{k}^{H}WAp_{k}}{\sum_{j\neq k}p_{j}^{H}A^{H}W^{H}h_{k}h_{k}^{H}WAp_{j}+\sigma_{k}^{2}}. \end{array}$$

Thus, the ergodic downlink rate of UT *k* can be stated as

(13) $$R_{k}=E_{H}\left\{\log_{2}\;\left(1+\Gamma_{k}\left(A,P\right)\right)\;\right\}.$$

There are two approximation approaches for the ergodic achievable rate (13) as follows.

1. **Upper bound approximation:** According to Jensen’s inequality and the concavity of $\log_{2}(\cdot )$, an upper bound of $R_{k}$ can be obtained as
  (14) $$\begin{array}{cc} {\bar{R}}_{k}≜ & \log_{2}\left(1+\frac{p_{k}^{H}A^{H}W^{H}E\left\{h_{k}h_{k}^{H}\right\}WAp_{k}}{\sum_{j\neq k}p_{j}^{H}A^{H}W^{H}E\left\{h_{k}h_{k}^{H}\right\}WAp_{j}+\sigma_{k}^{2}}\right)\\ = & \log_{2}\left(1+\frac{p_{k}^{H}{\hat{G}}_{k}p_{k}}{\sum_{j\neq k}p_{j}^{H}{\hat{G}}_{k}p_{j}+\sigma_{k}^{2}}\right), \end{array}$$
  where ${\hat{G}}_{k}=A^{H}W^{H}\Omega_{k}WA$.
2. **Lower bound approximation:** Let ${\bar{h}}_{k}=E\left\{h_{k}\right\}$. By treating ${\bar{h}}_{k}$ as the effective channel and regarding the random perturbation $\left(h_{k}-{\bar{h}}_{k}\right)$ as uncorrelated noise, a lower bound of $R_{k}$ can be derived as
  (15) $$\begin{array}{cc} {\tilde{R}}_{k}≜ & \log_{2}\left(1+\frac{p_{k}^{H}W^{H}A^{H}E\left\{h_{k}\right\}E\left\{h_{k}^{H}\right\}AWp_{k}}{\sum_{j=1}^{K}p_{j}^{H}W^{H}A^{H}E\left\{h_{k}h_{k}^{H}\right\}AWp_{j}-p_{k}^{H}W^{H}A^{H}E\left\{h_{k}\right\}E\left\{h_{k}^{H}\right\}AWp_{k}+\sigma_{k}^{2}}\right)\\ = & \log_{2}\left(1+\frac{|p_{k}^{H}{\bar{g}}_{k}{|}^{2}}{\sum_{j=1}^{K}p_{j}^{H}{\hat{G}}_{k}p_{j}-{\left|p_{k}^{H}{\bar{g}}_{k}\right|}^{2}+\sigma_{k}^{2}}\right), \end{array}$$
  where ${\bar{g}}_{k}=A^{H}W^{H}{\bar{h}}_{k}$.

Both approximations avoid taking expectations over nonlinear functions of the instantaneous channel vector $h_{k}$ in $R_{k}$. Intuitively, as the channel of UT *k* becomes more deterministic, i.e., as $\kappa_{k}$ increases, the expected channel tends to align more closely with the actual realization, leading both ${\bar{R}}_{k}$ and ${\tilde{R}}_{k}$ to approach $R_{k}$.

The main difference between the upper bound and lower bound approximations lies in how they treat the effective covariance matrix $h_{k}h_{k}^{H}$ of the *k*-th UT. The upper bound uses $E\{h_{k}h_{k}^{H}\}$, which includes the second-order statistics of both the LoS and scattered components, while the lower bound uses $E\left\{h_{k}\right\}E\left\{h_{k}^{H}\right\}$ that retains only the LoS component and ignores the contribution of the scattered paths. As a result, the lower bound tends to underestimate the effective channel gain compared to the upper bound.

**Figure 2:**
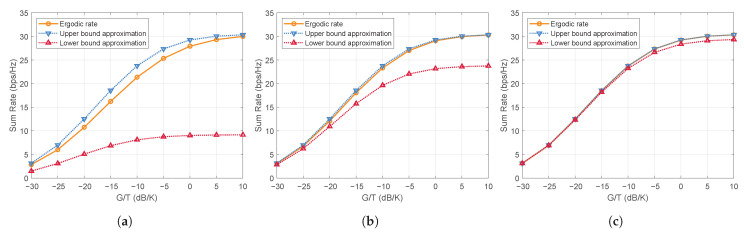
Tightness of rate bounds under different κ-factors: (**a**) κ=0 dB; (**b**) κ=10 dB; (**c**) κ=20 dB.

Figure 2 illustrates the accuracy of the two approximations under *κ* = 0, 10, and 20 dB, evaluated with beamspace ZF precoding. In the NLoS scenario of Figure 2a, the channel exhibits rich scattered components, causing the lower bound to deviate noticeably from the ergodic rate, whereas the upper bound remains much tighter. In the LoS-dominant case of Figure 2b, although the scattered components are weak, their second-order energy is still non-negligible. Hence, the upper bound approximation provides a more accurate representation of the actual channel power. When κ is large and the channel approaches a pure LoS condition in Figure 2c, the two approximations become close.

To obtain a tighter estimate of $R_{k}$, we adopt the upper bound approximation. Assuming a total transmit power constraint and a limited number of simultaneously activated beams, the WSR maximization problem is formulated as

(16) $$\begin{array}{cc} \max_{A,P}\; & \sum_{k=1}^{K}\beta_{k}\;{\bar{R}}_{k}\\ s.t.\; & \left(C1\right):\;\mathrm{Tr}\left[PP^{H}\right]\leq P_{T},\\ \; & \left(C2\right):\;\sum_{n=1}^{N}a_{n,b}\leq 1,\;\forall b,\\ \; & \left(C3\right):\;\sum_{b=1}^{B}a_{n,b}\leq 1,\;\forall n, \end{array}$$

where $\beta_{k}$ represents the weight of the *k*-th satellite UT.

The WSR maximization problem is inherently an NP-hard MINLP, and obtaining the global optimum of (A,P) is computationally prohibitive for LEO satellite systems with large-scale antenna arrays and limited on-board processing capability. To strike a balance between complexity and performance, we adopt the two-stage multibeam transmission approach.

## 3. Beamspace Transmission Design for Sum Rate Maximization Problem

### 3.1. Angle-Based Beam Selection Algorithm

In the fixed DFT codebook W, each codeword corresponds to a steering vector with a specific spatial direction. Once *B* mutually orthogonal codewords are selected, the UTs’ antenna-domain channels are effectively sampled along the corresponding spatial angles, mapping them from an N×K dimensional space to a B×K subspace, namely the beamspace domain. Prior studies have shown that as long as the selected subspace captures the majority of the channel power, the performance can approach that of the full-dimensional optimal design [27,28].

For the *n*-th codeword $w_{n}$ in the codebook W, its normalized beamforming gain toward UT *k* can be quantified by

(17) $$\eta_{k,n}={\left|v_{k}^{H}w_{n}\right|}^{2}.$$

**Proposition 1.**  (17) *can be reformulated as*

(18) $$\eta_{k,n}=\frac{1}{M^{2}}{\left|\frac{\sin\frac{\pi }{2}M_{x}\Delta \vartheta_{k,n}^{x}}{\sin\frac{\pi }{2}\Delta \vartheta_{k,n}^{x}}\right|}^{2}{\left|\frac{\sin\frac{\pi }{2}M_{y}\Delta \vartheta_{k,n}^{y}}{\sin\frac{\pi }{2}\Delta \vartheta_{k,n}^{y}}\right|}^{2}.$$

*where $\Delta \vartheta_{k,n}^{x}=\vartheta_{k}^{x}-\vartheta_{n}^{x}$ and $\Delta \vartheta_{k,n}^{y}=\vartheta_{k}^{y}-\vartheta_{n}^{y}$ denote the spatial angular differences between UT k and beam n, with $\left(\vartheta_{n}^{x},\vartheta_{n}^{y}\right)$ characterizing the 2D beam departure angles of the n-th codeword.*

**Proof.**  See Appendix A.    □

According to (17), computing $\eta_{k,n}$ for each UT–beam pair requires an inner product operation with a complexity of O(M). For LEO satellites equipped with large-scale antenna arrays, this operation becomes computationally expensive. However, by exploiting the 2D angular information as expressed in (18), the high-dimensional vector operations can be effectively avoided, since $\eta_{k,n}$ depends solely on the angular differences $\left(\Delta \vartheta_{k,n}^{x},\Delta \vartheta_{k,n}^{y}\right)$ and is independent of the absolute angular positions $\left(\vartheta_{k}^{x},\vartheta_{k}^{y}\right)$.

Define $N^{★}$ as the union of all selected codewords in the codebook, then the normalized beamspace channel power of UT *k* can be expressed as

(19) $$\eta_{k}=\sum_{\forall n\in N^{★}}\eta_{k,n}.$$

**Figure 3:**
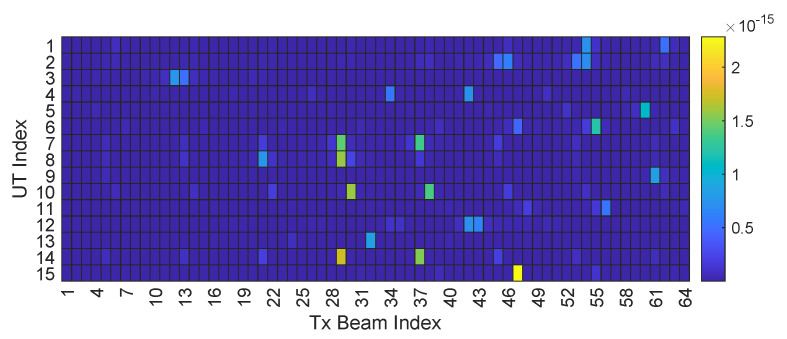
Sparsity of the beamspace channel.

To enhance the performance of beamspace precoding, the objective is to select codewords that can effectively improve ${\left\{\eta_{k}\right\}}_{\forall k}$ [29]. Yet, a larger $|N^{★}|$ does not necessarily lead to better performance due to the inherent sparsity of the beamspace channel, and may even incur higher computational complexity. Specifically, as the satellite channel is single-path dominated, the channel power of UT *k* tends to be concentrated around its LoS direction $\left(\vartheta_{k}^{x},\vartheta_{k}^{y}\right)$. Figure 3 illustrates the beamspace channel power sampled with an 8×8 DFT codebook. It can be observed that, for any given UT, most of its channel power is concentrated around a specific direction.

The sparsity of the beamspace channel simplifies the beam selection process by allowing the selection of only a small subset of beams to achieve near-optimal beamspace transmission performance. Moreover, this property enables decoupling the beam selection process for different UTs. Specifically, a lower bound of (20) can be formulated as

(20) $$\eta_{k}\geq \sum_{\forall n\in N_{k}^{★}}\eta_{k,n}≜{\tilde{\eta }}_{k},$$

where $N_{k}^{★}\subset N^{★}$ denotes the subset of selected codewords that are associated with beams pointing near the LoS direction of UT *k*. Since for UT *k*, the beams that can effectively improve $\eta_{k}$ are always distributed around its LoS direction, the lower bound in (20) is relatively tight. This implies that $N_{k}^{★}$ can be determined independently for each UT in practice, and the overall set of selected beams is obtained by taking the union over all UTs, i.e.,

(21) $$N^{★}=\underset{\forall k}{⋃}N_{k}^{★}\subset N.$$

To determine $N_{k}^{★}$, we first decompose the angular differences between UT *k* and beam *n* as

(22) $$\Delta \vartheta_{k,n}^{x}=\delta_{k}^{x}+\frac{2}{M_{x}}q_{k}^{x}\left(n\right),$$

(23) $$\Delta \vartheta_{k,n}^{y}=\delta_{k}^{y}+\frac{2}{M_{y}}q_{k}^{y}\left(n\right),$$

where $\delta_{k}^{x}=\vartheta_{k}^{x}-\vartheta_{\ell_{k}}^{x}\in \left[-\frac{1}{M_{x}},\frac{1}{M_{x}}\right]$ and $\delta_{k}^{y}=\vartheta_{k}^{y}-\vartheta_{\ell_{k}}^{y}\in \left[-\frac{1}{M_{y}},\frac{1}{M_{y}}\right]$ denote the space angle offsets between UT *k* and beam $\ell_{k}$, $\ell_{k}\in N$ is the index of the beam closest to $\left(\vartheta_{k}^{x},\vartheta_{k}^{y}\right)$. $q_{k}^{x}\left(n\right)\in Z$ and $q_{k}^{y}\left(n\right)\in Z$ are integers representing the distance between beam *n* and $\ell_{k}$. For a given pair $\left(\delta_{k}^{x},\delta_{k}^{y}\right)$, sorting ${\left\{\eta_{k,n}\right\}}_{\forall n}$ in descending order yields the following sequences of $q_{k}^{x}\left(n\right)$ and $q_{k}^{y}\left(n\right)$:

(24) $$T^{b}=\left\{\begin{array}{cc} \{0,-1,1,-2,2,\ldots \}, & \delta_{k}^{b}\geq 0\\ \{0,1,-1,2,-2,\ldots \}, & \delta_{k}^{b}<0 \end{array}\right.,b\in \left\{x,y\right\}.$$

**Figure 4:**
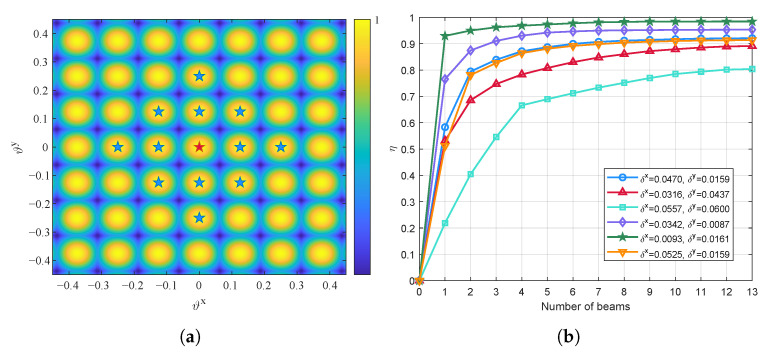
Illustration of the number of beams and the corresponding normalized beamspace channel power: (**a**) Beam set with $\left(\vartheta_{\ell_{k}}^{x},\vartheta_{\ell_{k}}^{y}\right)=\left(0,0\right)$, $|N_{k}|=13$; (**b**) $\eta_{k}$ vs. $|N_{k}^{★}|$.

For UT *k*, by selecting the first few elements from $T^{b}$, a subset of codewords concentrated around $\ell_{k}$ can be constructed, denoted as $N_{k}$. Then, $N_{k}^{★}$ can be obtained by selecting elements only from $N_{k}$ rather than from the entire codebook, which further reduces the computational complexity. As illustrated in Figure 4, for six random realizations of $\left(\delta_{k}^{x},\delta_{k}^{y}\right)$ with $\left(\vartheta_{\ell_{k}}^{x},\vartheta_{\ell_{k}}^{y}\right)=\left(0,0\right)$ and $|N_{k}|=13$, $\eta_{k}$ rapidly increases with the number of selected beams $|N_{k}^{★}|$, and near-optimal channel sampling can be achieved by selecting only 1–4 beams. Accordingly, we define a simple rule for constructing $N_{k}^{★}$ as

(25) $$N_{k}^{★}=\left\{n\in N_{k}:\eta_{k,n}\geq \alpha_{k}\eta_{k,\ell_{k}}\right\},$$

where $\eta_{k,\ell_{k}}$ denotes the normalized beamspace channel power captured by the beam closest to the LoS direction of UT *k* and $\alpha_{k}$ is the threshold.

The beam selection results directly determine the beam selection matrix A as

(26) $$a_{n,b}=\left\{\begin{array}{cc} 1, & \mathrm{if}\;n\in N^{★},\\ 0, & \mathrm{otherwise}. \end{array}\right.$$

The above angle-based beam selection algorithm is summarized in Algorithm 1.
**Algorithm 1** Angle-based Beam Selection Algorithm**Input:** ${\left\{\gamma_{k},\theta_{k},\alpha_{k},|N_{k}|\right\}}_{\forall k}$, **W**, *B*.
  1: **Initialize:**   1-a: $\left(\vartheta_{k}^{x},\vartheta_{k}^{y}\right)\leftarrow \theta_{k}$, ∀k∈K.   1-b: $\left(\vartheta_{n}^{x},\vartheta_{n}^{y}\right)\leftarrow w_{n}$, ∀n∈N.  2: **for** 
k∈K **do**  3:      $\ell_{k}\leftarrow \arg\min_{n\in N}\left({\left|\Delta \vartheta_{k,n}^{x}\right|}^{2}+{\left|\Delta \vartheta_{k,n}^{y}\right|}^{2}\right)$.  4:      Calculate $\eta_{k,\ell_{k}}$ as described in (18).  5:      Set $N_{k}$ with indices $\ell_{k}$ and sequences $T^{x},T^{y}$.  6:      **for** $n\in N_{k}$ **do**  7:          Calculate $\eta_{k,n}$ as described in (18).  8:      **end for**  9:      $N_{k}^{★}\leftarrow \left\{\;n\in N_{k}|\eta_{k,n}\geq \alpha_{k}\;\eta_{k,\ell_{k}}\;\right\}$.
10: **end for**
11: $N^{★}\leftarrow {⋃}_{k=1}^{K}N_{k}^{★}$
12: **if** $|N^{★}|>B$ **then**
13:      **for** $n\in N^{★}$ **do**
14:           $s_{n}\leftarrow \sum_{k=1}^{K}\eta_{k,n}$.
15:      **end for**
16:      $N^{★}\leftarrow {arg max}_{S\subseteq N^{★},\;\left|S\right|=B}\;\sum_{n\in S}s_{n}$.
17: **end if**
18: Calculate A as described in (26).
**Output:**
A.


### 3.2. Beamspace WMMSE Precoding

Once the beamspace channel is determined, LP is required to suppress multibeam interference. Among various LP strategies, the WMMSE precoding algorithm is particularly appealing as it is mathematically equivalent to the WSR maximization problem and guarantees convergence to a stationary point with favorable performance.

To transform the WSR maximization problem in (16) into an equivalent WMMSE problem, we first perform covariance decomposition on $\Omega_{k}$ as

(27) $$\Omega_{k}=q_{k}q_{k}^{H},$$

where $q_{k}=\sqrt{\gamma_{k}}v_{k}$.

Then the WMMSE problem can be given as

(28) $$\begin{array}{cc} \min_{M,U,P} & \sum_{\forall k}\beta_{k}\left(m_{k}e_{k}-\log\left(m_{k}\right)\right)\\ s.t. & (C1),(C2),(C3), \end{array}$$

where $M=\mathrm{diag}(m_{1},m_{2},\ldots ,m_{k})≻0$ denotes the WMMSE weight matrix. $U=\mathrm{diag}(u_{1},u_{2},\ldots ,u_{K})$ represents the virtual receiver with $u_{k}$ corresponding to the receive filter of UT *k*. $e_{k}$ is denoted by

(29) $$e_{k}=E\{{\left|{\hat{s}}_{k}-s_{k}\right|}^{2}\}.$$

where ${\hat{s}}_{k}$ denotes a linear estimate of $s_{k}$

(30) $$\begin{array}{c} {\hat{s}}_{k}=u_{k}{\hat{g}}_{k}^{H}\sum_{\forall j}p_{j}s_{j}+u_{k}n_{k}, \end{array}$$

where ${\hat{g}}_{k}=A^{H}W^{H}q_{k}$.

By substituting (30) into (29), the MSE of UT *k* can be rewritten as

(31) $$\begin{array}{c} e_{k}=E\{{\left|u_{k}{\hat{g}}_{k}^{H}p_{k}-1\right|}^{2}\}+\sum_{j\neq k}{\left|u_{k}\right|}^{2}E\{{\left|{\hat{g}}_{k}^{H}p_{j}\right|}^{2}\}+{\left|u_{k}\right|}^{2}\sigma_{k}^{2}. \end{array}$$

**Proposition 2.** 
*The WSR maximization problem in* (16) *is equivalent to the WMMSE problem in* (28), *in the sense that both formulations yield the same optimal precoder P with the same beam selection matrix A.*

**Proof.**  See Appendix B.    □

Different from conventional WMMSE formulations that rely on iCSI, our WMMSE problem is formulated directly on sCSI $\Omega_{k}$, which ensures robustness and low overhead for transmission implementation.

Although the WMMSE problem is not jointly convex, it is convex with respect to each variable when the others are fixed. Hence, an alternating optimization strategy can be employed. The update steps are as follows.

With M and P fixed, the optimal $u_{k}$ is obtained by minimizing $e_{k}$ with respect to $u_{k}$, yielding

(32) $$u_{k}^{★}=\frac{p_{k}^{H}{\hat{g}}_{k}}{\sum_{j=1}^{K}p_{j}^{H}{\hat{g}}_{k}{\hat{g}}_{k}^{H}p_{j}+\sigma_{k}^{2}}.$$

Based on such an expression, the corresponding optimal value of $e_{k}$ can be computed as

(33) $$e_{k}^{★}=\frac{\sum_{j\neq k}{\left|p_{j}^{H}{\hat{g}}_{k}\right|}^{2}+\sigma_{k}^{2}}{\sum_{j=1}^{k}{\left|p_{j}^{H}{\hat{g}}_{k}\right|}^{2}+\sigma_{k}^{2}}.$$

With U and P fixed, the optimal WMMSE weight is given by $m_{k}^{★}={\left(e_{k}^{★}\right)}^{-1}$, which can be written as

(34) $$m_{k}^{★}=1+\frac{p_{k}^{H}{\hat{g}}_{k}{\hat{g}}_{k}^{H}p_{k}}{\sum_{j\neq k}p_{j}^{H}{\hat{g}}_{k}{\hat{g}}_{k}^{H}p_{j}+\sigma_{k}^{2}}.$$

With M and U fixed, the optimization problem in terms of P reduces to

(35) $$\begin{array}{cc} \min_{P} & \sum_{\forall k}\beta_{k}m_{k}e_{k}\\ s.t. & (C1), \end{array}$$

whose Lagrangian function can be derived as

(36) $$L\left(P,\mu \right)=\sum_{\forall k}\beta_{k}m_{k}e_{k}\left(P\right)+\mu \left(\mathrm{Tr}\left[PP^{H}\right]-P_{T}\right),$$

where μ is the Lagrange multiplier. By setting $\frac{\partial L(P,\mu )}{\partial p_{k}^{H}}=0$, we formulate the closed-form iterative expression for the precoding vector of UT *k* as

(37) $$p_{k}^{★}\left(\mu \right)={\left(\sum_{j=1}^{K}\beta_{j}m_{j}{\left|u_{j}\right|}^{2}{\hat{g}}_{j}{\hat{g}}_{j}^{H}+\mu I\right)}^{-1}\beta_{k}m_{k}u_{k}^{*}{\hat{g}}_{k}.$$

For brevity, we combine the precoding vectors of all satellite UTs as

(38) $$P^{★}\left(\mu \right)=\eta {\left(\hat{\Omega }+\mu I\right)}^{-1}D,$$

where $\hat{\Omega }=\sum_{j=1}^{K}\beta_{j}m_{j}{\left|u_{j}\right|}^{2}{\hat{g}}_{j}{\hat{g}}_{j}^{H}$, $d_{k}=\beta_{k}m_{k}u_{k}^{*}{\hat{g}}_{k}$, and $D=[d_{1},d_{2},\ldots ,d_{K}]$. The scaling factor η ensures the power constraint and is given by

(39) $$\eta \left(\mu \right)=\sqrt{\frac{P_{T}}{\mathrm{Tr}[D^{H}{\left(\hat{\Omega }+\mu I\right)}^{-2}D]}}.$$

Finally, by substituting $P^{★}\left(\mu \right)$ into (35) and differentiating with respect to μ, the closed-form solution is obtained as

(40) $$\mu =\frac{\sum_{\forall k}\beta_{k}m_{k}{\left|u_{k}\right|}^{2}\sigma_{k}^{2}}{P_{T}}.$$

The above beamspace WMMSE precoding algorithm is summarized in Algorithm 2.
**Algorithm 2** Beamspace WMMSE Precoding**Input:** ${\left\{{\hat{g}}_{k},\gamma_{k},\sigma_{k},\beta_{k}\right\}}_{\forall k}$, $P_{T}$, $I_{\max}$, χ.  1: **Initialize:**   1-a: i←0.   1-b: ${\bar{R}}_{k}\leftarrow 0$, ∀k∈K.  2: **while** $i<I_{\max}$ **do**  3:      i←i+1.  4:      ${\bar{R}}_{k}^{\prime }\leftarrow {\bar{R}}_{k}$, ∀k∈K.  5:      Update $u_{k}^{★}$ as in (32).  6:      Update $m_{k}^{★}$ as in (34).  7:      Update $p_{k}^{★}$ as in (37).  8:      Calculate ${\bar{R}}_{k}$ as described in (14).  9:      **if** $\frac{\sum_{k=1}^{K}\beta_{k}\left({\bar{R}}_{k}-{\bar{R}}_{k}^{\prime }\right)}{\sum_{k=1}^{K}\beta_{k}{\bar{R}}_{k}^{\prime }}\leq \chi$ **then**
10:         **break**
11:      **end if**
12: **end while**
**Output:** 
P.


## 4. Simulation Results

We consider a broadband LEO satellite equipped with a UPA. Multiple beams are generated under an FFR setting to simultaneously serve multiple UTs randomly distributed within the satellite’s circular coverage region. Each UT is assumed to be a single-antenna handheld device. The simulations are conducted in the L/S frequency band, which is widely adopted in mobile satellite communications for voice and data services to handheld terminals. The key simulation parameters are summarized in Table 1.

In this section, we mainly compare the following multibeam transmission schemes:

- **DFTBF**: A baseline that selects *K* beams from the fixed DFT codebook W that best matches the UTs’ LoS directions. This method has the lowest computational complexity but does not incorporate LP methods for interference mitigation, thus serving as a performance lower bound.
- **sWMMSE**: An sCSI-based antenna-domain WMMSE precoding scheme applied directly to the full channel, serving as the performance upper bound.
- **sBWMMSE-ABS**: An sCSI-based beamspace WMMSE precoding scheme that first selects *B* beams from W based on the UTs’ angular information (Algorithm 1), and then performs WMMSE precoding in the beam domain (Algorithm 2).
- **sBWMMSE-1B**: A simplified scheme that selects a single LoS-matched beam for each UT, followed by sCSI-based beamspace WMMSE precoding.

Monte Carlo simulations are performed to evaluate the performance of different transmission schemes. In each trial, *K* UTs are uniformly placed within the satellite footprint. Since $\eta_{k,n}$ in (18) depends only on the angular differences between UT *k* and beam *n* (i.e., $\left(\Delta \vartheta_{k,n}^{x},\Delta \vartheta_{k,n}^{y}\right)$) rather than the absolute space angles of UT *k* (i.e., $\left(\vartheta_{k}^{x},\vartheta_{k}^{y}\right)$), the threshold in Algorithm 1 can be chosen identically for all UTs; that is, we set $\alpha_{k}=\alpha$ for all UTs. Additionally, it is assumed that all UTs share the same G/T, Rician factor $\kappa_{k}$, and noise power $\sigma_{k}$, i.e., $\kappa_{k}=\kappa ,\;\sigma_{k}=\sigma ,\;\forall k$. The channels are generated according to (9), and the link gain of UT *k* is obtained from the link budget $\gamma_{k}=\frac{P_{T}G_{T}G_{R}Mc^{2}}{{\left(4\pi fL_{k}\right)}^{2}}$, where $G_{T}$ and $G_{R}$ represent the antenna gain of the satellite and UTs, $L_{k}$ is the distance between the satellite and UT *k* [31].

**Figure 5:**
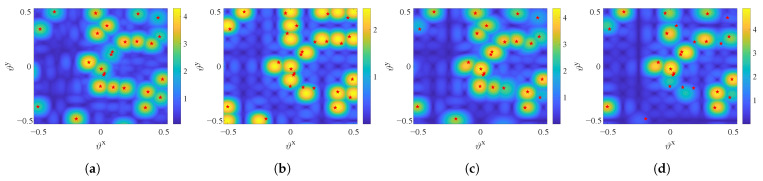
Satellite beam patterns, α=0.2, M=256, G/T = −30 dB/K, K=50: (**a**) sWMMSE; (**b**) DFTBF; (**c**) sBWMMSE-ABS; (**d**) sBWMMSE-1B.

Figure 5 compares the beam patterns of the four schemes. The sWMMSE scheme produces the most accurate mainlobes and achieves deep nulls in non-service directions, thereby providing the best multibeam transmission performance. The DFTBF scheme relies solely on fixed codeword-based beamforming without LP methods, thus resulting in a significant discrepancy between the mainlobe directions and the UTs’ LoS angles, as well as strong sidelobe interference. Furthermore, as different UTs may be assigned the same best-matching beam, it is difficult to support full-frequency reuse across beams. sBWMMSE-1B alleviates some of these problems by applying WMMSE precoding on the selected beams, resulting in improved interference control, but residual misalignment and sidelobe leakage remain. sBWMMSE-ABS achieves beam patterns close to sWMMSE, while notably improving mainlobe accuracy and reducing sidelobe interference compared with DFTBF and sBWMMSE-1B, which stems from the enlarged beam set that enables improved accuracy of channel sampling and provides extra degrees of freedom for interference mitigation.

**Figure 6:**
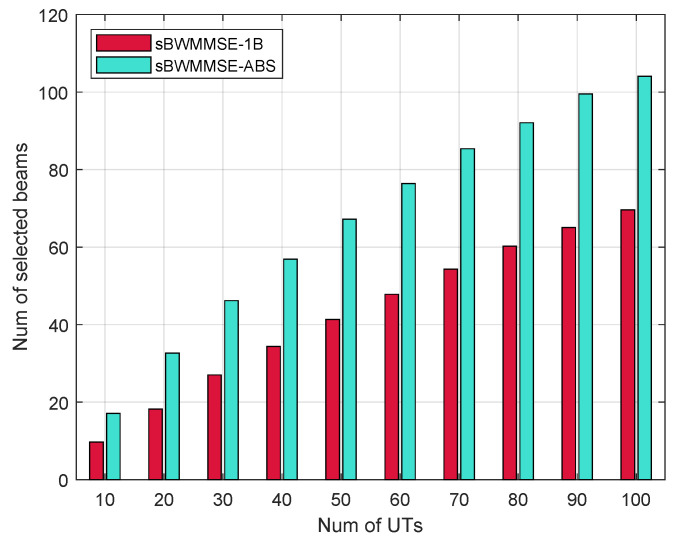
$|N^{★}|$ vs. *K*, α=0.2, M=256, G/T = −30 dB/K.

Figure 6 shows the variation in the selected beam count with the number of UTs for sBWMMSE-ABS and sBWMMSE-1B schemes. As *K* increases, $|N^{★}|$ grows sub-linearly, since UTs located closely in the angular domain often share common optimal or near-optimal beams. This effect becomes more pronounced with higher UT density, thereby slowing the growth of the union set $N^{★}={⋃}_{\forall k}N_{k}^{★}$. Furthermore, although sBWMMSE-ABS introduces auxiliary beams pointing near each UT’s LoS direction, the increase in $|N^{★}|$ compared with sBWMMSE-1B remains modest and well below the size of the full codebook.

**Figure 7:**
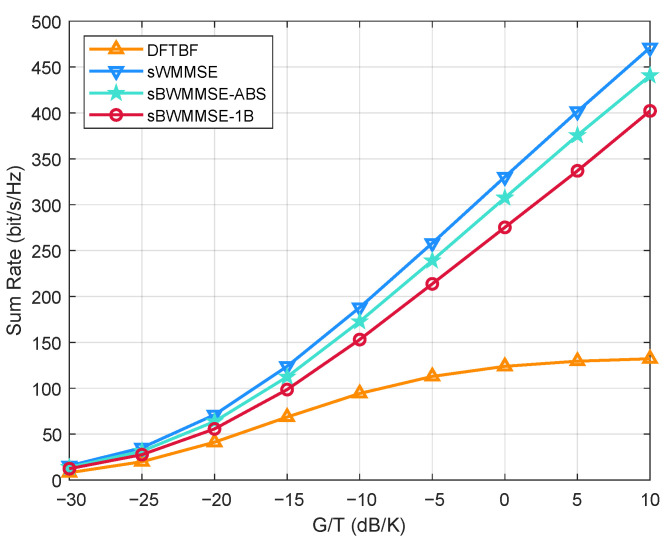
Average sum rate vs. G/T, α=0.2, M=256, K=40.

Figure 7 presents the average sum rate performance of four schemes with respect to the UT G/T values. As G/T increases, the system transitions from a noise-limited to an interference-limited regime. The DFTBF scheme quickly saturates due to its inability to suppress interference, serving as the lower bound. sWMMSE consistently delivers the best performance and acts as the upper bound. Both beamspace schemes, sBWMMSE-ABS and sBWMMSE-1B, achieve substantial gains over DFTBF. Among them, sBWMMSE-ABS exhibits performance much closer to the sWMMSE solution, demonstrating the effectiveness of the proposed beam selection (Algorithm 1) and precoding (Algorithm 2) strategies.

**Figure 8:**
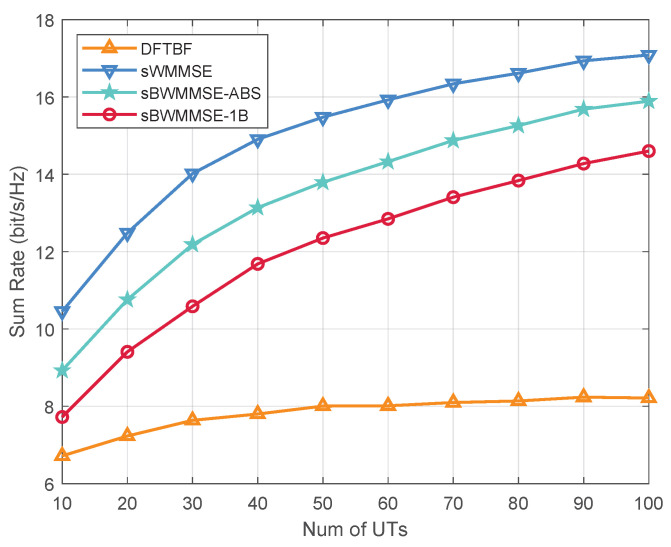
Average sum rate vs. *K*, α=0.2, M=256, G/T = −30 dB/K.

In Figure 8, we investigate the impact of the number of UTs on these schemes. For the simplest DFTBF scheme, the system sum rate increases with the number of UTs *K*, but the growth gradually slows down. When K≥50, the sum rate saturates and remains almost constant thereafter. Benefiting from the interference suppression capability of WMMSE precoding, the sWMMSE, sBWMMSE-ABS and sBWMMSE-1B schemes all significantly outperform DFTBF, and continue to achieve performance gains as *K* increases. Among them, the sWMMSE scheme consistently provides the highest sum rate across the entire range, while sBWMMSE-ABS outperforms sBWMMSE-1B and remains close to sWMMSE in most cases.

**Figure 9:**
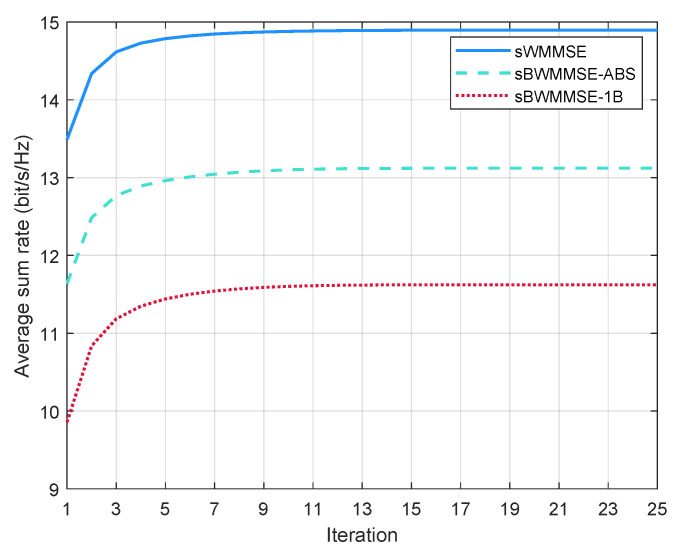
Convergence of sWMMSE, sBWMMSE-ABS, and SBMMSE.

For the iterative algorithms sWMMSE, sBWMMSE-ABS, and sBWMMSE-1B, we study their convergence behaviors in sum rate. As illustrated in Figure 9, the sum rate performances of all the three schemes quickly converge within the specified number of iterations.

Table 2 compares the computational complexity of the considered algorithms. For DFTBF, the primary computational burden arises from identifying the best-matching beam for each UT within the codebook, which results in a complexity of O(KN). For the sWMMSE scheme, the computational complexity per iteration can be expressed as $O(KM^{2}+M^{3}+M^{2}K)$, among which the $O(M^{3})$ term dominates when M≫K. Therefore, the overall complexity is characterized by $O(I_{\max}M^{3})$, with the lower-order terms omitted. The complexity of sBWMMSE-ABS consists of two parts: beam selection based on Algorithm 1 and beamspace WMMSE precoding based on Algorithm 2. In the first part, the complexity is $O(KN+K|N_{k}|)$; in the second part, the analysis is similar to that of sWMMSE precoding, but on a reduced dimension, leading to $O(I_{\max}B^{3})$, where the beam set has a size of $|N^{★}|\leq B$. The complexity of sBWMMSE-1B is similar to that of sBWMMSE-ABS, except that the beam selection process is simplified: each UT selects only its best-matching beam, resulting in a beam set size $|N^{★}|\leq K$. Consequently, the overall complexity of sBWMMSE-1B is $O(KN+I_{\max}K^{3})$. Comparing the computational complexity of the four schemes, DFTBF is the most lightweight with its cost grows approximately linearly with the codebook (and thus array) size. By contrast, sWMMSE has the highest complexity, scaling cubically with the array size. By operating on a dimension-reduced beamspace channel, sBWMMSE-ABS and sBWMMSE-1B substantially reduce the computational burden, placing their complexity between DFTBF and sWMMSE.

**Figure 10:**
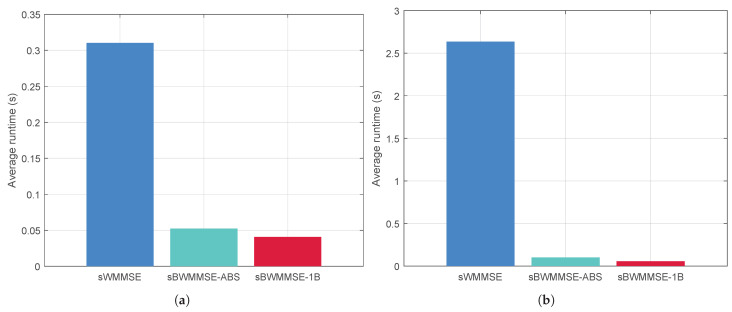
Running time comparison: (**a**) M=256; (**b**) M=1024.

The runtime results in Figure 10 further corroborate these findings. sWMMSE exhibits the highest runtime and shows strong sensitivity to *M*, consistent with its cubic scaling $O(M^{3})$. sBWMMSE-1B and sBWMMSE-ABS achieve substantially lower runtime, where sBWMMSE-ABS incurs slightly higher runtime than sBWMMSE-1B due to B≥K. Considering that the sum rate performance of sBWMMSE-ABS is close to that of sWMMSE, these results confirm that sBWMMSE-ABS achieves a favorable trade-off between performance and computational complexity.

**Figure 11:**
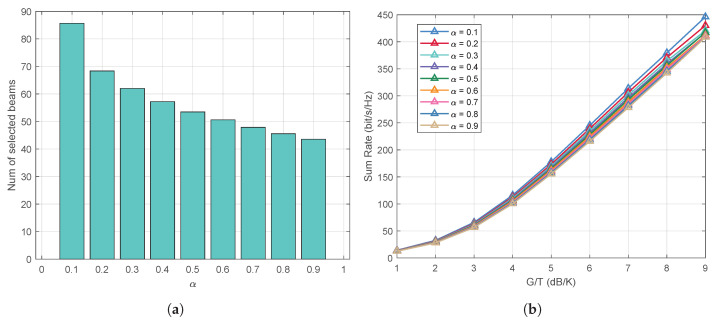
Effect of the threshold parameter α on the performance of sBWMMSE-ABS: (**a**) $|N^{★}|$ vs. α, K=50, M=256, G/T = −30 dB/K.; (**b**) Average sum rate vs. α, K=50, M=256.

In Figure 11, we study the impact of the threshold parameter α on the performance of the proposed algorithm sBWMMSE-ABS. As illustrated in Figure 11a, when α decreases, the number of selected beams $|N^{★}|$ increases, and vice versa. This brings two opposite effects. On one hand, as $|N^{★}|$ increases, the projection of the antenna-domain channel energy onto the beam region increases, which is also illustrated in Figure 4b. Accordingly, the effectiveness of beamspace precoding is enhanced, leading to an increase in system sum rate, as shown in Figure 11b. On the other hand, as $|N^{★}|$ increases, the dimension of the beamspace channel matrix and the beamspace linear precoding matrix also increases, which results in higher computational complexity.

## 5. Conclusions

This paper investigated an sCSI-based beamspace transmission design for LEO satellite communication systems. We first derived an sCSI-based upper bound for the ergodic sum rate and used it to formulate the corresponding WSR maximization problem. Then, we proposed an angle-based beam selection algorithm, which utilized the proportion of channel power captured by each beam as the selection metric and exploited the LoS-dominant nature of satellite channels to reduce computational complexity. Based on the low-dimensional beamspace channel, we transformed the WSR problem into an equivalent sWMMSE problem and derived a beamspace precoding scheme to suppress CCI. Simulation results demonstrated that the proposed scheme achieved performance close to antenna-domain precoding while effectively reducing computational complexity. Moreover, since the proposed beamspace transmission framework relied only on slowly varying sCSI, it successfully mitigated the challenge of acquiring iCSI.

## Figures and Tables

**Table 1:** Simulation parameters [30].

Parameter	Value
Orbit altitude	785.41 km
Transmit antenna size	16×16/32×32
Maximum transmit power	300 W
Gain of TX antennas	0 dBi
Gain of RX antennas	0 dBi
UT antenna noise temperature	290 K
UT G/T	[−30,−10] dB/K
Number of UTs	10–70
Distribution of UTs	Uniform
Carrier frequency	2 GHz
System bandwidth	20 MHz

**Table 2:** Computational complexity.

Methods	Complexity Order
DFTBF	$O\;\left(KN\right)$
sWMMSE	$O\;\left(I_{\max}\;M^{3}\right)$
sBWMMSE-1B	$O\;\left(KN+I_{\max}K^{3}\right)$
sBWMMSE-ABS	$O\;\left(KN+I_{\max}\;B^{3}\right)$

## Data Availability

Data is contained within the article.

## Abbreviations

The following abbreviations are used in this manuscript:

LEO	Low Earth orbit
MEO	Medium Earth orbit
GEO	Geostationary orbit
UT	User terminal
OBP	On-board processor
CoMSat	Coordinated multiple-satellite
CCI	Co-channel interference
SE	Spectral efficiency
CSI	Channel state information
iCSI	Instantaneous channel state information
sCSI	Statistical channel state information
AoD	Angle of departure
LoS	Line-of-sight
MIMO	Multiple-input multiple-output
mMIMO	Massive multiple-input multiple-output
UPA	Uniform planar array
DBF	Digital beamforming
DFT	Discrete Fourier transform
FFT	Fast Fourier transform
FFR	Full-frequency reuse
MSE	Mean square error
LP	Linear precoding
WSR	Weighted sum rate
WMMSE	Weighted minimum mean square error
sWMMSE	sCSI-based weighted minimum mean square error
MF	Matched filter
ZF	Zero-forcing
RZF	Regularized zero-forcing
SINR	Signal-to-interference-plus-noise ratio
SLNR	Signal-to-leakage-plus-noise ratio

## Appendix A. Proof of Proposition 1

With a UPA of size $M_{x}\times M_{y}$, the array response of UT *k* can be separated into two different components in the *x*-axis and *y*-axis:

(A1) $$v_{k}=v_{k}^{x}\left(\vartheta_{k}^{x}\right)⊗v_{k}^{y}\left(\vartheta_{k}^{y}\right).$$

Similarly, each codeword in the beamforming codebook associated with the $M_{x}\times M_{y}$ UPA can also be factorized as the Kronecker product of two one-dimensional DFT vectors, i.e.,

(A2) $$w_{n}=w_{n}^{x}\left(\vartheta_{n}^{x}\right)⊗w_{n}^{y}\left(\vartheta_{n}^{y}\right).$$

Here, $w_{n}^{b}\left(\vartheta_{n}^{b}\right)\left(b\in \left\{x,y\right\}\right)$ share the same functional form with $v_{k}^{b}\left(\vartheta_{k}^{b}\right)$ and can be written as

(A3) $$w_{n}^{b}=\frac{1}{\sqrt{M_{b}}}{\left[\;1,\;e^{-j\frac{2\pi }{\lambda }d_{b}\vartheta_{n}^{b}},\;\ldots ,\;e^{-j\frac{2\pi }{\lambda }d_{b}\left(M_{b}-1\right)\vartheta_{n}^{b}}\;\right]}^{T}.$$

Using ${\left(a⊗b\right)}^{H}\left(c⊗d\right)=\left(a^{H}c\right)⊗\left(b^{H}d\right)$, (17) can be reformulated as

(A4) $$\eta_{k,n}={\left|{\left(v_{k}^{x}\right)}^{H}w_{n}^{x}\right|}^{2}{\left|{\left(v_{k}^{y}\right)}^{H}w_{n}^{y}\right|}^{2}.$$

For each dimension b∈{x,y}, the inner product of $v_{k}^{b}$ and $w_{n}^{b}$ forms a finite geometric sum and can be computed as

(A5) $$\begin{array}{cc} {\left(v_{k}^{b}\right)}^{H}w_{n}^{b}= & \frac{1}{M_{b}}\sum_{m=0}^{M_{b}-1}e^{-jm\psi_{b}}\\ = & \frac{1}{M_{b}}\cdot \frac{1-e^{jM_{b}\psi_{b}}}{1-e^{j\psi_{b}}}, \end{array}$$

where $\psi_{b}≜\frac{2\pi }{\lambda }d_{b}\left(\vartheta_{k}^{b}-\vartheta_{n}^{b}\right)$ denote the phase increments.

By multiplying the numerator and denominator by $e^{-jM_{b}\psi_{b}/2}$ and $e^{-j\psi_{b}/2}$, (A5) can be rewritten in a sine form as

(A6) $${\left(v_{k}^{b}\right)}^{H}w_{n}^{b}=\frac{1}{M_{b}}\;e^{-j\frac{\left(M_{b}-1\right)\psi_{b}}{2}}\;\frac{\sin\;\left(\frac{M_{b}\psi_{b}}{2}\right)}{\sin\;\left(\frac{\psi_{b}}{2}\right)},$$

Accordingly, we obtain

(A7) $${\left|{\left(v_{k}^{b}\right)}^{H}w_{n}^{b}\right|}^{2}=\frac{1}{M_{b}^{2}}{\left|\frac{\sin\;\left(\frac{M_{b}\psi_{b}}{2}\right)}{\sin\;\left(\frac{\psi_{b}}{2}\right)}\right|}^{2}.$$

Therefore, the fraction of captured channel power can be written as

(A8) $$\eta_{k,n}=\frac{1}{M_{x}^{2}}{\left|\frac{\sin\;\left(\frac{M_{x}\psi_{x}}{2}\right)}{\sin\;\left(\frac{\psi_{x}}{2}\right)}\right|}^{2}\cdot \frac{1}{M_{y}^{2}}{\left|\frac{\sin\;\left(\frac{M_{y}\psi_{y}}{2}\right)}{\sin\;\left(\frac{\psi_{y}}{2}\right)}\right|}^{2}.$$

When $d_{x}=d_{y}=\lambda /2$, (A8) reduces to (18).

This completes the proof.

## Appendix B. Proof of Proposition 2

By successively substituting the optimal $u_{k}^{★}$ in (32) and $m_{k}^{★}$ in (34) into the objective function of the WMMSE problem (28), the cost can be reformulated as a function depending only on (P). With $m_{k}^{★}={\left(e_{k}^{★}\right)}^{-1}$, we obtain

(A9) $$\begin{array}{c} m_{k}^{★}e_{k}^{★}-\log\left(m_{k}^{★}\right)=1-\log\;\left(1+\frac{|p_{k}^{H}{\hat{g}}_{k}{|}^{2}}{\sum_{j\neq k}{\left|p_{j}^{H}{\hat{g}}_{k}\right|}^{2}+\sigma_{k}^{2}}\right). \end{array}$$

Therefore, the objective of the WMMSE problem (28) can be rewritten as

(A10) $$\begin{array}{cc} \; & \sum_{k=1}^{K}\beta_{k}\left(m_{k}^{★}e_{k}^{★}-\log\left(m_{k}^{★}\right)\right)\\ \; & =\sum_{k=1}^{K}\beta_{k}-\sum_{k=1}^{K}\beta_{k}\log\left(1+\frac{|p_{k}^{H}{\hat{g}}_{k}{|}^{2}}{\sum_{j\neq k}{\left|p_{j}^{H}{\hat{g}}_{k}\right|}^{2}+\sigma_{k}^{2}}\right)\\ \; & =\sum_{k=1}^{K}\beta_{k}-\sum_{k=1}^{K}\beta_{k}{\bar{R}}_{k} \end{array}$$

Since $\sum_{k=1}^{K}\beta_{k}$ is a constant independent of (P), minimizing the WMMSE objective is equivalent to maximizing the WSR objective in (16). Hence, the two problems yield the same optimal precoder P and beam selection matrix A.

This completes the proof.
